# Amelioration of 5-Fluorouracil–Induced Hepatorenal Toxicity by Epigallocatechin Gallate–Functionalized Selenium Nanoparticles: A Multi-Targeted Protective Approach

**DOI:** 10.3390/ijms27093887

**Published:** 2026-04-27

**Authors:** Wesam Abd El-Fattah, Ahlem Guesmi, Naoufel Ben Hamadi, Hani S. Hafez, Mohamed A. Ali, Khaled M. Alam-ElDein, Mohamed H. A. Gadelmawla

**Affiliations:** 1 Chemistry Department, College of Science, Imam Mohammad Ibn Saud Islamic University (IMSIU), P.O. Box 5701, Riyadh 11432, Saudi Arabia; 2 Department of Zoology, Faculty of Science, Suez University, Suez 43533, Egypt; 3 Pharmaceutical Biotechnology Department, School of Biotechnology, Badr University in Cairo, Cairo 11829, Egypt; 4 Molecular Biology and Biotechnology Department, School of Biotechnology, Badr University in Cairo, Cairo 11829, Egypt; 5 Life Sciences Department, Faculty of Biotechnology, Sinai University, Kantara Branch, Ismailia 41636, Egypt

**Keywords:** epigallocatechin-3-gallate, selenium, nanoparticle, hepatotoxicity, renal toxicity, 5-flourouracil

## Abstract

5-Fluorouracil (5-FU) is a cornerstone chemotherapeutic agent that is extensively utilized in the management of malignancies; however, its clinical utility is constrained by its narrow therapeutic index and dose-limiting toxicities. The study aimed to study the hepato-nephroprotective effects of epigallocatechin gallate (EGCG) and EGCG mediated selenium nanoparticles and their effect in mitigating the toxicity induced by 5-FU. EGCG-functionalized selenium nanoparticles (EGCG-SeNPs) were produced by mixing sodium selenite, with EGCG acting as both the reducing and stabilizing agent. Nanoparticles were characterized using UV-vis spectroscopy, FT-IR, dynamic light scattering, zeta potential analysis, and transmission electron microscopy. 35 adult rats were randomly assigned to control, 5-FU, 5-FU + Na_2_SeO_3_, 5-FU + EGCG, and 5-FU + EGCG-SeNPs groups. Hepatorenal toxicity was induced by intraperitoneal 5-FU administration during the final five days of the experiment. Serum biochemical markers, tissue oxidative stress, antioxidant enzyme, inflammatory cytokine levels, and apoptosis-related gene expression were evaluated. Immunohistochemical analysis of Nrf2 and Keap1 and histopathological examination of tissues were performed. 5-FU induced severe hepatorenal toxicity, evidenced by marked elevations in liver and kidney function biomarkers, excessive oxidative stress, inflammatory cytokine overproduction, NF-κB activation, and apoptotic signaling. Treatment with EGCG-SeNPs markedly ameliorated 5-FU-induced hepatic and renal dysfunction, restoring liver enzyme and kidney biomarker levels to near-normal levels more effectively than EGCG or sodium selenite alone. EGCG-SeNPs significantly suppressed lipid peroxidation, NGAL, and inflammatory mediators while robustly enhancing antioxidant defenses and activating the Nrf2/HO-1 pathway with concomitant Keap-1 downregulation, strongly inhibited NF-κB signaling, normalized cytokine balance, reduced poly (ADP-ribose) (PAR) activation, and attenuated apoptosis. EGCG–SeNPs confer superior protection against 5-FU–induced hepatorenal toxicity compared to EGCG or inorganic selenium alone. The potent protective effects of EGCG–SeNPs are mediated through coordinated antioxidant, anti-inflammatory, and anti-apoptotic mechanisms, primarily via activation of the Nrf2/HO-1 axis and suppression of NF-κB signaling.

## 1. Introduction

The classical antimetabolite 5-Fluorouracil (5-FU) is a synthetic fluorinated pyrimidine analog that is extensively used as a chemotherapeutic agent. It exerts cytotoxic effects by disrupting nucleic acid metabolism, hindering DNA and RNA synthesis. This interference prevents replication and transcription in normal and cancer cells. The drug is metabolized by the liver, with minimal unchanged drug excretion by the kidneys [1]. Clinically, it remains a cornerstone agent, extensively used in the treatment of colorectal, breast, gastrointestinal, head and neck, and pancreatic cancers [2]. Despite its therapeutic efficacy, the clinical value of the drug is limited by a restricted therapeutic index and dose-limiting effects [3]. Notably, 5-FU has been implicated in the development of nephrotoxicity and hepatotoxicity, characterized by elevated hepatic enzyme activity as well as renal biomarkers, reflecting significant hepato-renal burden and systemic adverse effects associated with its prolonged or high-dose exposure [4].

An expanding corpus of contemporary research has underscored the therapeutic potential of bioactive natural compounds in alleviating adverse effects associated with conventional chemotherapeutic regimens [5]. Among these agents, epigallocatechin gallate (EGCG), the predominant catechin in green tea, has emerged as a powerful antioxidant and cytoprotective molecule with multifaceted biological activities. EGCG exerts its protective influence primarily by modulating intracellular redox homeostasis, suppressing inflammatory mediators, and augmenting the production and activity of pivotal antioxidant enzymes including superoxide dismutase (SOD), catalase (CAT), and glutathione peroxidase (GPx) [6,7].

Selenium (Se), an indispensable micronutrient incorporated into several selenoproteins, has the potential to preserve redox equilibrium and mitigate oxidative stress (OS)-induced cellular injury [8]. However, the therapeutic efficacy of selenium is often limited by its narrow margin between physiological necessity and toxicity. To overcome this limitation, selenium nanoparticles (SeNPs) have been developed as a next-generation delivery platform, offering superior bioavailability, targeted delivery, and reduced systemic toxicity compared to conventional Se forms [9]. Moreover, extensive experimental evidence indicates that SeNPs confer pronounced hepatoprotective and nephroprotective effects by attenuating oxidative stress, downregulating proinflammatory signaling cascades, and promoting cellular regeneration [10].

Despite known protective effects of EGCG and selenium against chemotherapy-induced toxicity, their clinical use is limited by poor bioavailability and narrow safety margins. Although selenium nanoparticles (SeNPs) offer improved delivery, the combined efficacy of EGCG-functionalized SeNPs remains insufficiently explored in vivo. Moreover, their comparative advantage over EGCG or inorganic selenium alone and the precise molecular mechanisms particularly involving Nrf2/HO-1 and NF-κB pathways are not well established.

This study aimed to explore the hepatoprotective and nephroprotective effects of EGCG-functionalized selenium nanoparticles (EGCG-SeNPs) and their potential to counteract the toxicity induced by the chemotherapeutic agent 5-fluorouracil (5-FU). It assessed how EGCG-SeNPs could alleviate OS, inflammation, and apoptosis associated with 5-FU treatment, thereby enhancing liver and kidney function and offering a protective mechanism against the adverse effects of chemotherapy.

## 2. Results

### 2.1. Characterization of EGCG-SeNPs

#### 2.1.1. Zeta Size and Zeta Potential Analysis

**Figure 1:**
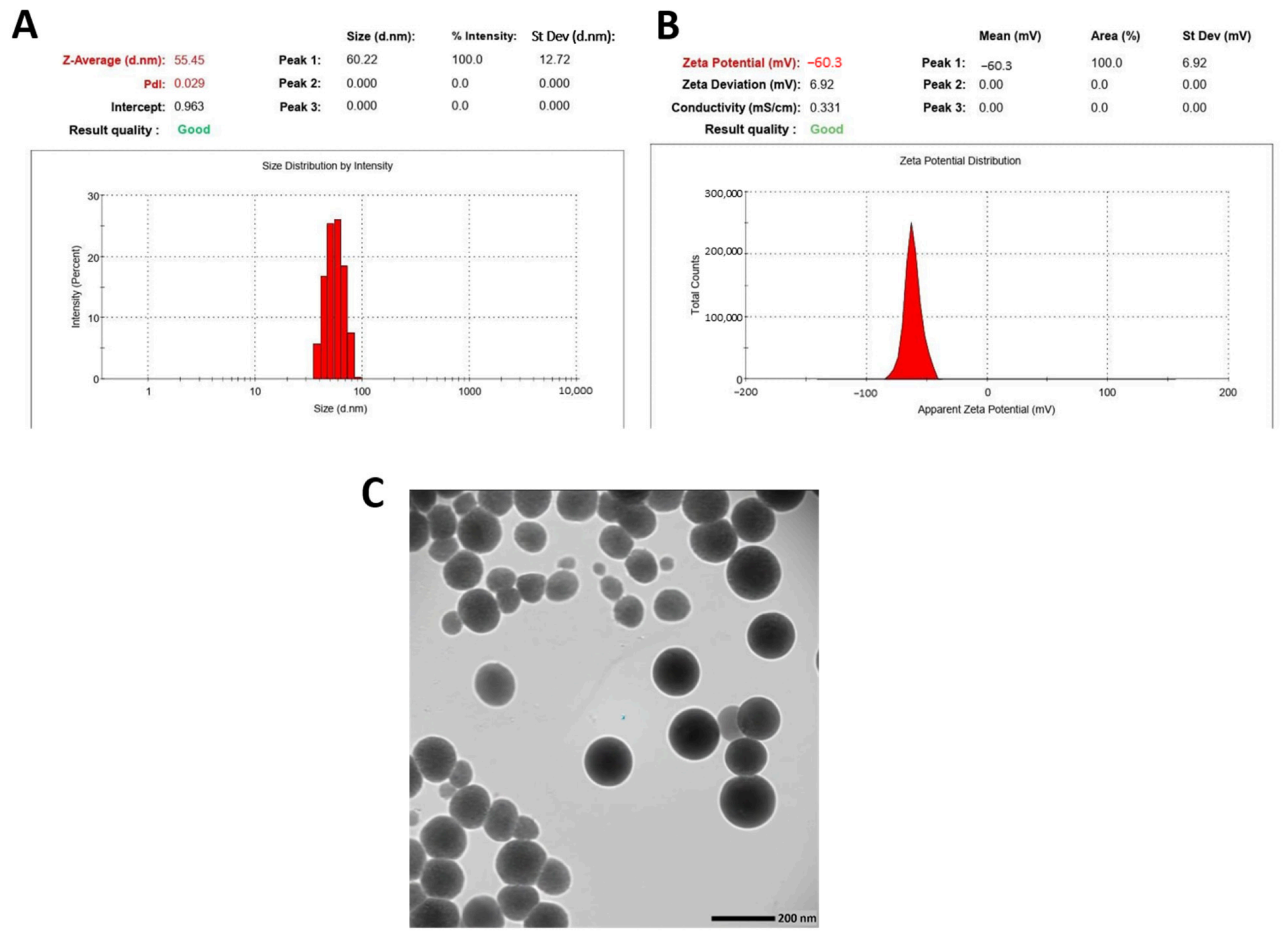
(**A**) Zeta size, (**B**) zeta potential, and (**C**) TEM of EGCG-SeNPs revealing successful formulation (scale bar = 200 nm).

DLS analysis showed that the EGCG-mediated SeNPs were within the nanoscale range, with a narrow size distribution reflecting controlled particle growth. The hydrodynamic diameter confirmed the presence of stable colloidal nanoparticles, whereas the zeta potential values indicated surface charge, promoting particle repulsion. This charge is a result of EGCG molecules adsorbed onto the nanoparticle surface, enhancing stability and limiting aggregation. The data demonstrate the role of EGCG in stabilizing SeNPs and maintaining suspensions (Figure 1).

#### 2.1.2. TEM Analysis

TEM imaging further corroborated the successful formation of EGCG-SeNPs, revealing spherical particles with a relatively uniform morphology. The nanoparticles were consistently observed within the nanoscale dimension, with particle sizes mainly distributed between 30 and 60 nm (Figure 1), confirming agreement with the size analysis results. Although the particles were generally well dispersed across the examined fields, minimal clustering was detected, likely resulting from the inherently high surface energy of EGCG-SeNPs. Despite this limited aggregation, the overall morphological features indicate that EGCG effectively functioned as both a capping and reducing agent, yielding SeNPs with controlled geometry, homogeneous size distribution, and stable nanoscale characteristics.

#### 2.1.3. UV–Visible Spectroscopic Characterization

**Figure 2:**
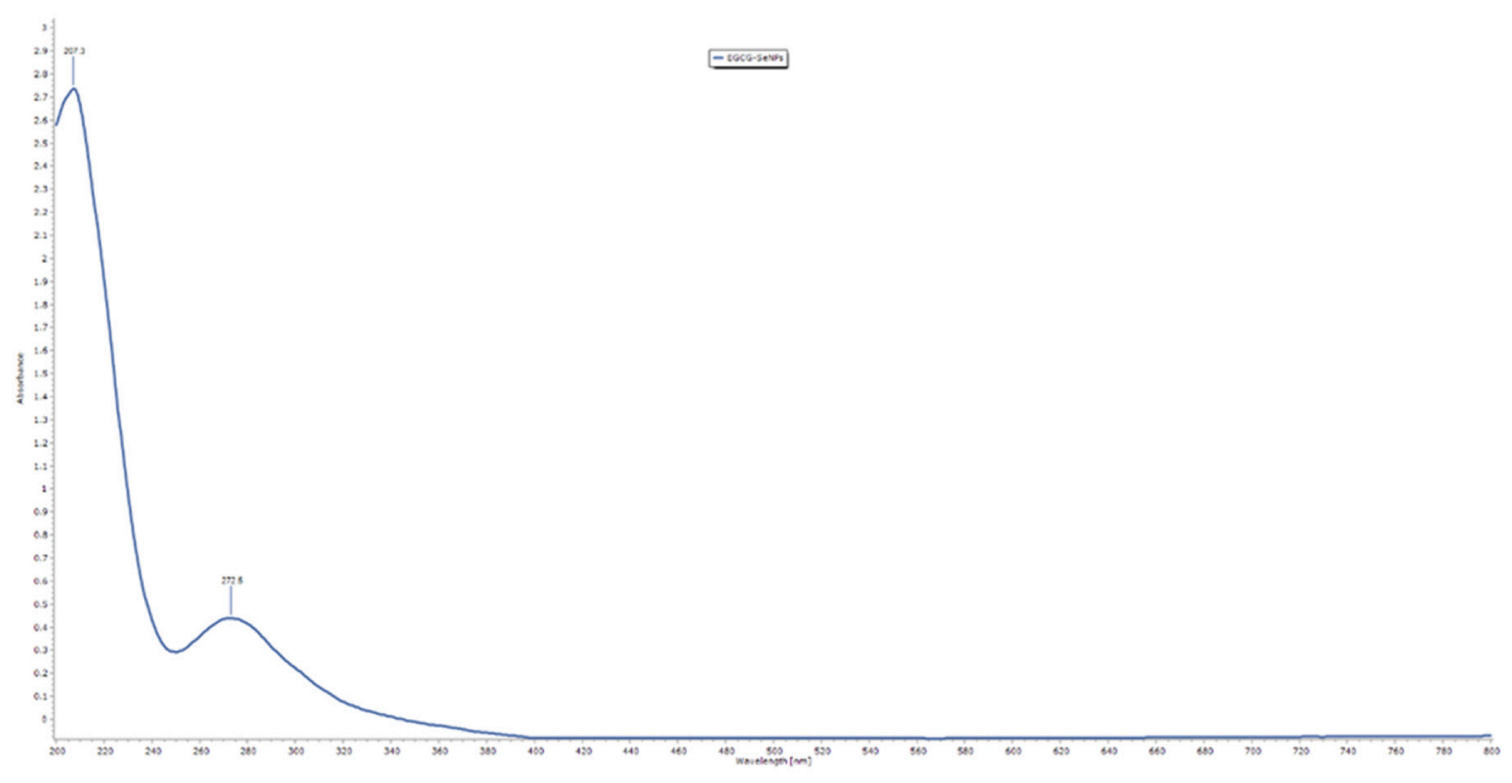
UV-vis spectroscopy of EGCG-SeNPs, demonstrating successful formulation.

Ultraviolet–visible (UV–Vis) spectroscopic analysis was employed to confirm the successful availability and stability of EGCG-mediated selenium nanoparticles. The absorption spectrum of the synthesized EGCG-SeNPs displayed a distinct and intense absorption band in the UV region, with a prominent peak centered at approximately 210 nm and a secondary absorption shoulder observed near 272 nm (Figure 2). The strong absorption at approximately 210 nm is attributed to π → π* electronic transitions of the aromatic rings present in EGCG, whereas the secondary band at approximately 272 nm is characteristic of n → π* transitions correlated with phenolic functional groups. These spectral features provide clear evidence of the involvement of EGCG molecules in the reduction and stabilization of Se ions during NP formation.

Notably, no additional absorption peaks were observed <300 nm, along with a gradual decrease in absorbance throughout the visible region, indicating the formation of well-dispersed selenium nanoparticles with minimal aggregation. Such spectral behavior is consistent with stable nanoscale systems and suggests effective capping of the nanoparticle surface by EGCG, preventing particle growth and agglomeration.

#### 2.1.4. FT-IR Spectroscopic, and XRD Characterization

The FTIR spectra of EGCG and EGCG mediated selenium nanoparticles (EGCG-SeNPs) exhibited distinct spectral differences, demonstrating the successful formation of the nanoparticles. The native EGCG spectrum displayed a broad absorption band at 3548 cm^−1^, resulting from O–H stretching vibrations of the abundant phenolic hydroxyl groups, along with a C–H stretch at approximately 2985 cm^−1^. The prominent peak at approximately 1611 cm^−1^ originated from aromatic C=C stretching, while the absorption bands extending from 1346 to 1044 cm^−1^ corresponded to C-O stretching vibrations of the phenolic and ether bonds present in the flavonoid structure.

**Figure 3:**
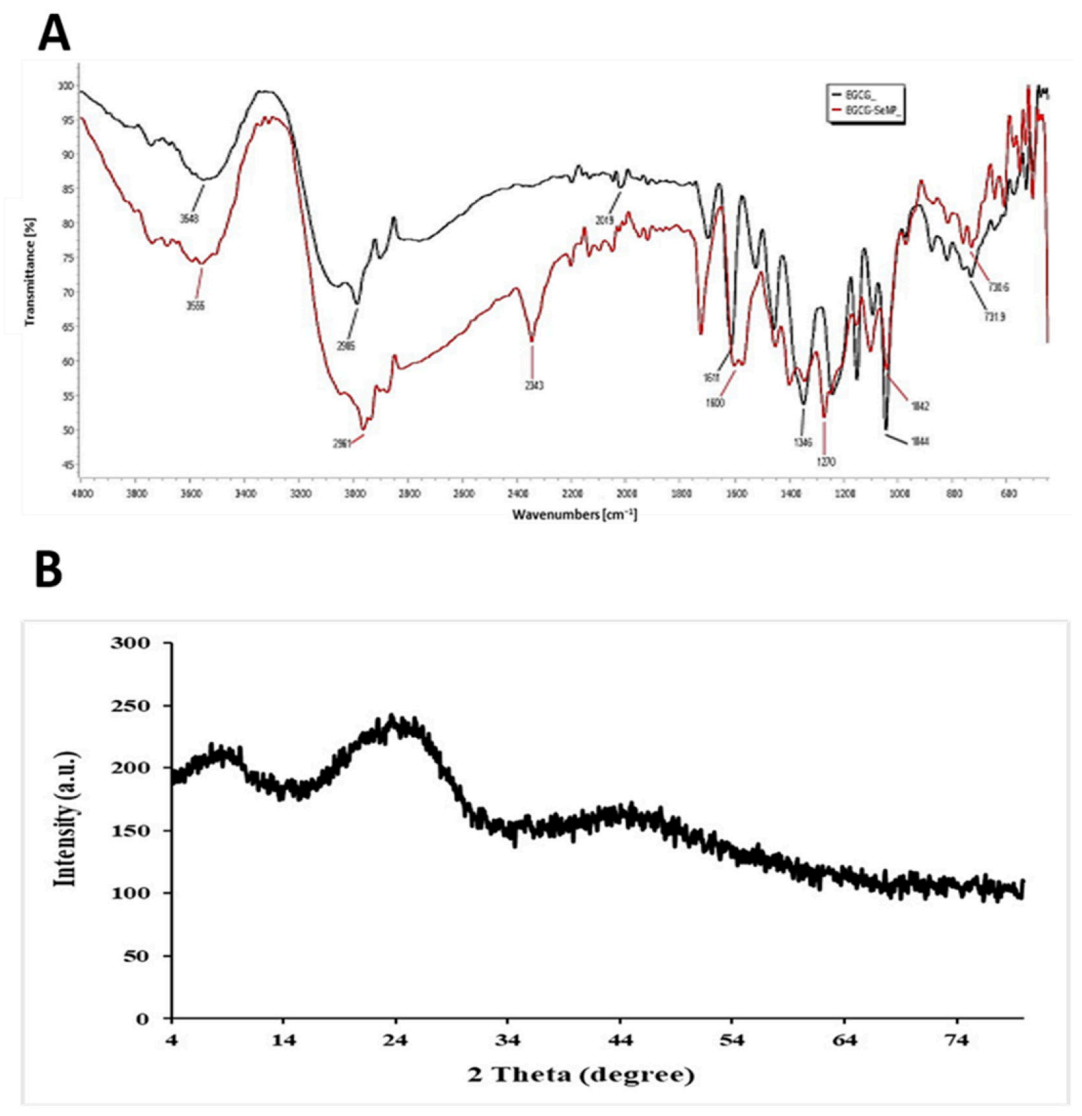
(**A**) FTIR spectrogram of EGCG and EGCG mediated selenium nanoparticles (EGCG-SeNPs), (**B**) XRD of EGCG mediated selenium nanoparticles (EGCG-SeNPs).

These characteristic bands exhibited observable changes in the intensities and movements of the O–H (3555 cm^−1^), C–H (2961 cm^−1^), and aromatic (1600 cm^−1^) peaks in the EGCG-SeNPs spectrum upon nanoparticle synthesis, including changes in positions and intensities and alterations in the fingerprint region (1270–1042 cm^−1^). These spectral changes indicate the active role of EGCG functional groups in the decrease in selenium ions and the subsequent stabilization of the obtained nanoparticles, as shown in Figure 3A.

X-ray diffraction (XRD) analysis was performed to evaluate the structural characteristics of the synthesized EGCG-SeNPs. The diffraction pattern exhibited broad and diffused peaks rather than sharp Bragg reflections, indicating the predominantly amorphous or poorly crystalline nature of the nanoparticles.

A broad hump was observed in the region of approximately 20–30° (2θ), which is characteristic of nanoscale selenium structures stabilized by organic capping agents such as EGCG. The absence of distinct, sharp diffraction peaks suggests that the nanoparticles possess reduced crystallinity, which is commonly reported for biogenic or polyphenol-mediated selenium nanoparticles, as shown in Figure 3B.

### 2.2. Biological Activity of Epigallocatechin Gallate-Functionalized Selenium Nanoparticles

#### 2.2.1. Liver Enzyme Biomarkers

**Figure 4:**
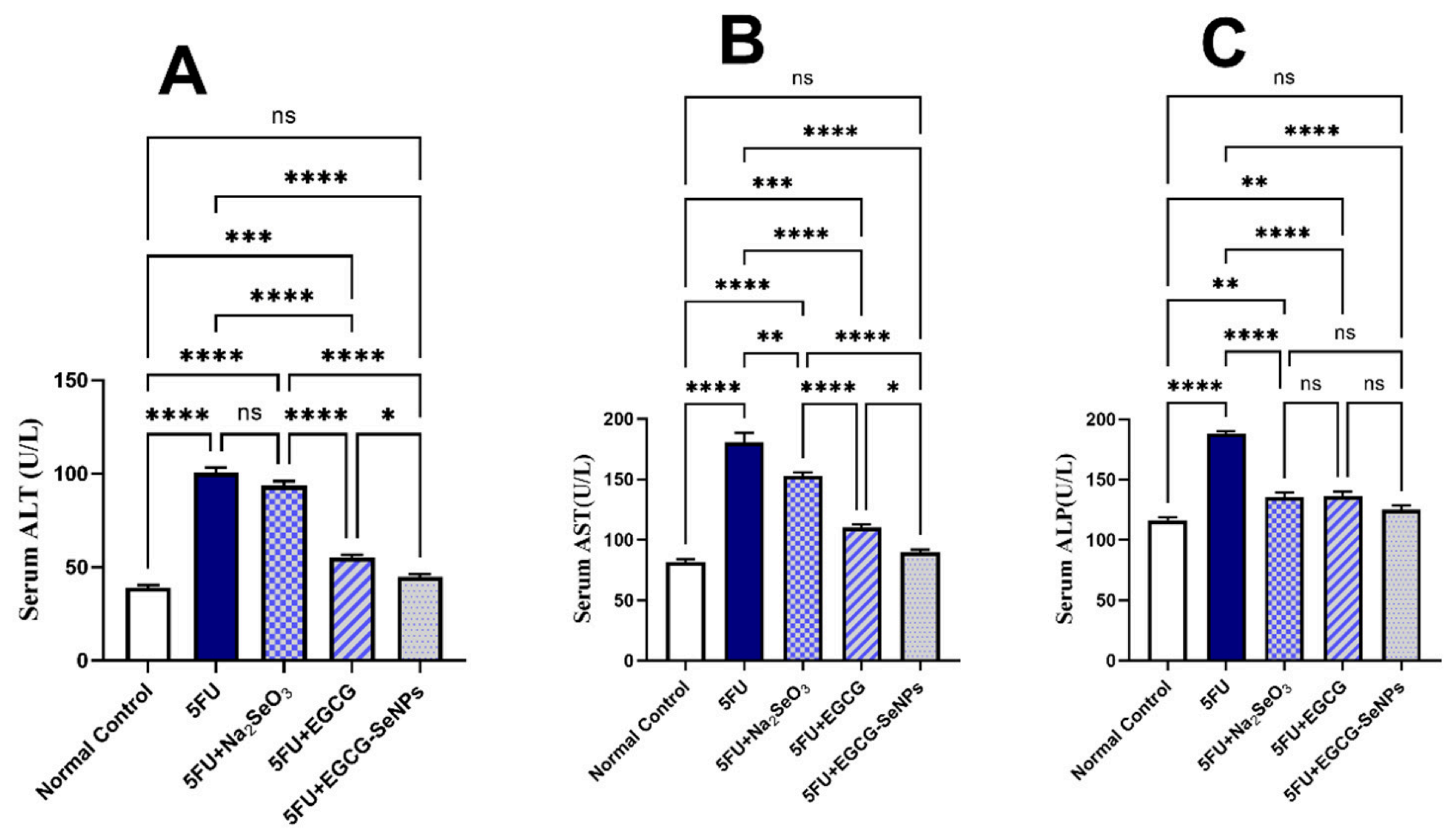
Impact of treatments on hepatic function enzymes in 5-FU-induced hepatotoxicity. Serum activities of (**A**) ALT, (**B**) AST, and (**C**) ALP, reflecting hepatocellular integrity and biliary function. Na_2_SeO_3_, EGCG, and EGCG-SeNPs treatments attenuated enzyme elevations induced by 5-FU. Data are expressed as mean ± SEM (*n* = 7). Statistical significance among groups is indicated by asterisks; *: *p* < 0.05, **: *p* < 0.005, ***:*p* < 0.0005, ****: *p* < 0.00005, ns: non-significant.

As illustrated in Figure 4, 5-fluorouracil (5-FU) administration elicited a pronounced hepatotoxic response, as reflected by significant elevations in serum liver enzyme activities. Compared to the normal control group, 5-FU-treated rats exhibited marked increases in alanine aminotransferase (ALT), aspartate aminotransferase (AST), and alkaline phosphatase (ALP) by 188.57%, 159.52%, and 183.33%, respectively (*p* < 0.05), confirming severe hepatocellular injury, enhanced membrane permeability, and biliary dysfunction induced by 5-FU.

Co-treatment with sodium selenite (5FU + Na_2_SeO_3_) resulted in limited amelioration of hepatic enzyme disturbances. Relative to the normal control group, serum ALT, AST, and ALP levels remained significantly elevated by 162.86%, 109.52%, and 177.78%, respectively; *p* < 0.05. When compared with the 5-FU group, these changes represented non-remarkable reductions by 8.91%, 19.27%, and 1.96% for ALT, AST, and ALP, respectively, indicating a modest hepatoprotective effect of inorganic selenium.

In contrast, EGCG supplementation (5FU + EGCG) significantly attenuated 5-FU-induced hepatic injury. Compared with the 5-FU group, serum ALT, AST, and ALP activities were substantially reduced by 42.57%, 53.21%, and 46.08%, respectively (*p* < 0.05). However, enzyme levels in this group remained substantially higher than those in the NC, showing residual elevations by 65.71%, 21.43%, and 52.78% for ALT, AST, and ALP, respectively.

Remarkably, treatment with 5FU + EGCG-SeNPs produced the most pronounced hepatoprotective effect. Compared to the 5-FU group, ALT, AST, and ALP activities declined substantially by 61.39%, 55.96%, and 55.88%, respectively *p* < 0.05. Relative to the control group, enzyme activities exhibited only minimal, non-pathological increases by 11.43%, 14.29%, and 25.00% for ALT, AST, and ALP, respectively. Moreover, enzyme values in the EGCG-SeNPs-treated group were substantially lower than those observed in all other treated groups *p* < 0.05, showing substantial recovery towards normal physiological values.

#### 2.2.2. Kidney Function Biomarkers (Urea, Creatinine, Cystatin-C, and KIM-1)

**Figure 5:**
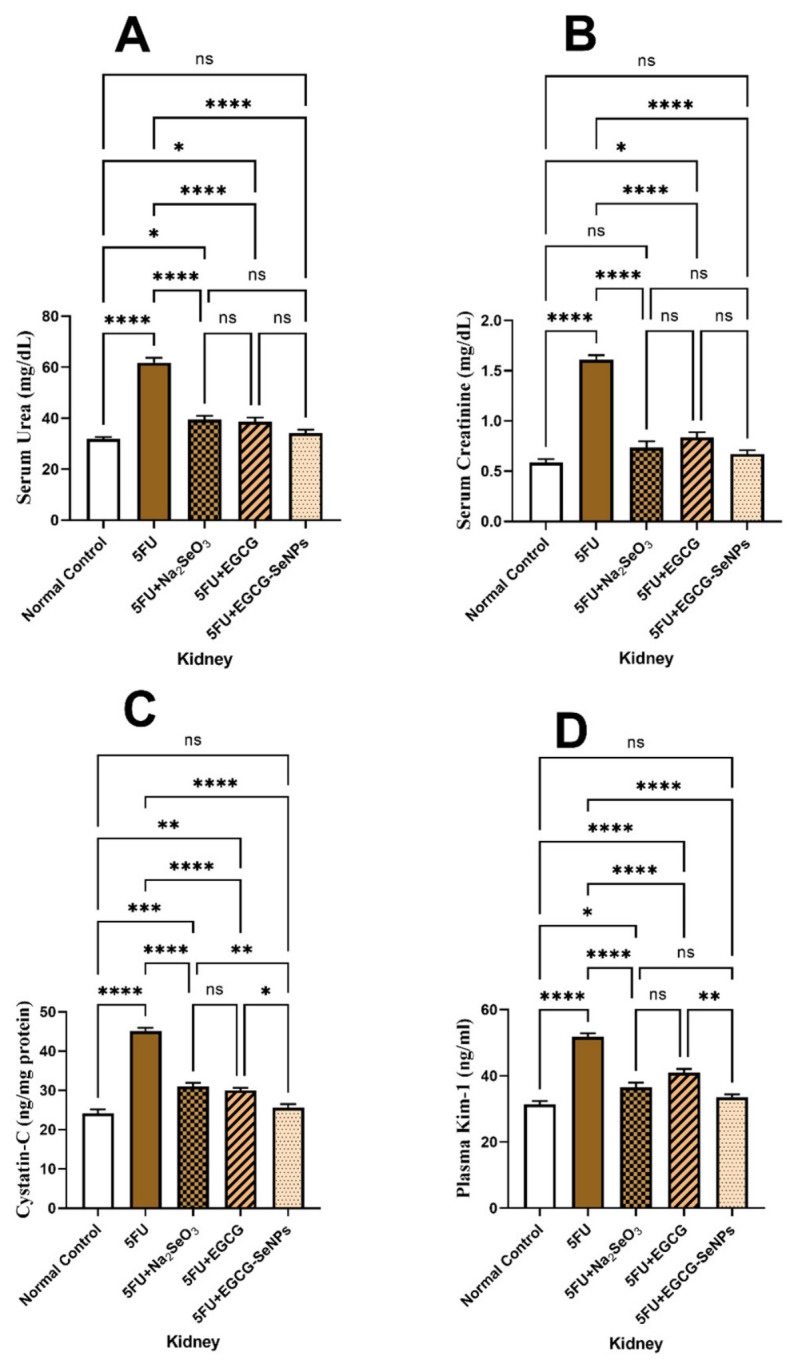
Effect of treatments on kidney function biomarkers in 5-FU-induced hepato-renal toxicity. Serum levels of (**A**) urea, (**B**) creatinine, (**C**) cystatin-C, and (**D**) KIM-1, reflecting renal functional status. Na_2_SeO_3_, EGCG, and EGCG-SeNPs treatments ameliorated 5-FU-induced renal dysfunction. Data are expressed as the mean ± SEM (*n* = 7). Statistical significance among groups is indicated by asterisks; *: *p* < 0.05, **: *p* < 0.005, ***: *p* < 0.0005, ****: *p* < 0.00005, ns: non-significant.

As illustrated in Figure 5, 5-FU administration provoked a pronounced nephrotoxic response, as evidenced by significant elevations in serum renal function biomarkers. Compared to the normal group, 5-FU-treated rats exhibited remarkable increases in urea, creatinine, cystatin-C, and kidney injury molecule-1 (KIM-1) levels by 127.59%, 100%, 61.76%, and 90.91%, respectively; *p* < 0.05, confirming severe renal functional impairment, reduced glomerular filtration capacity, and enhanced tubular injury.

Co-treatment with sodium selenite (5FU + Na_2_SeO_3_) partially attenuated 5-FU-induced renal dysfunction. Relative to the normal control group, serum urea, creatinine, cystatin-C, and KIM-1 levels remained significantly elevated by 41.38%, 9.38%, 17.65%, and 33.33%, respectively; *p* < 0.05. When compared with the 5-FU group, these changes represented nonsignificant reductions, indicating limited nephroprotective efficacy of inorganic selenium against 5-FU-induced renal injury.

In contrast, EGCG supplementation (5FU + EGCG) significantly mitigated renal dysfunction induced by 5-FU. Compared to the 5-FU group, serum urea, creatinine, cystatin-C, and KIM-1 levels were significantly reduced by 31.03%, 6.25%, 8.82%, and 21.21%, respectively (*p* < 0.05). Nevertheless, biomarker levels in this group remained significantly elevated relative to the normal control group, showing residual increases by 31.03%, 6.25%, 8.82%, and 21.21%, respectively.

Remarkably, treatment with 5FU + EGCG-SeNPs produced the most pronounced nephroprotective effect. Compared to the 5-FU group, urea, creatinine, cystatin-C, and KIM-1 levels declined significantly by 50.00%, 9.38%, 27.45%, and 41.67%, respectively; *p* < 0.05. Relative to the control group, kidney biomarkers exhibited minimal changes by 13.79%, 9.38%, 11.76%, and 15.15%, respectively. Moreover, biomarker levels in the EGCG-SeNPs-treated group were substantially lower than those observed in all other treated groups, *p* < 0.05, showing substantial recovery towards normal physiological values.

#### 2.2.3. Oxidative and Stress Response Markers in Hepatic and Renal Tissues

(MDA, NGAL, GSH, SOD, CAT, GR, HO-1, and Nrf2)

**Figure 6:**
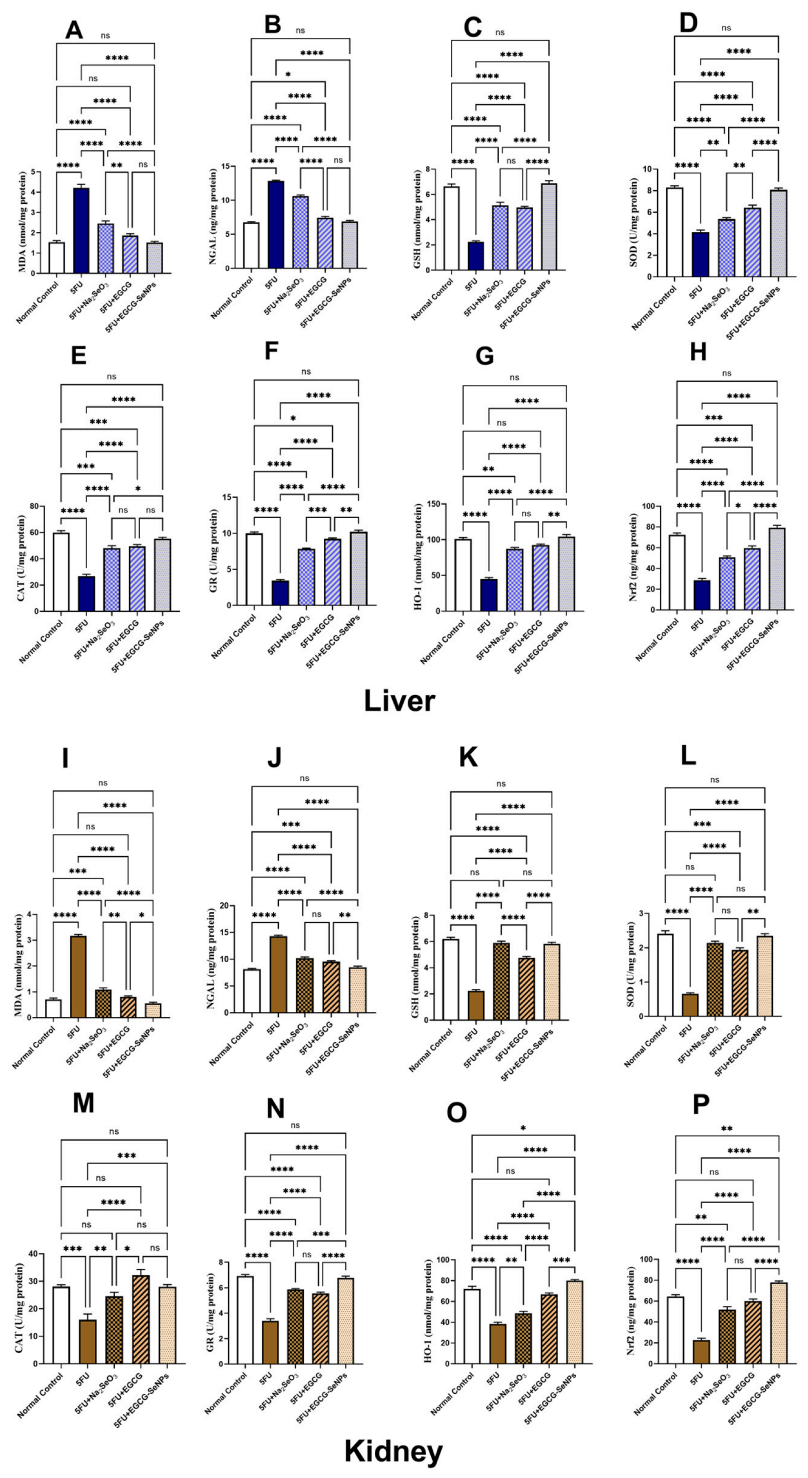
Effects of EGCG, sodium selenite, and EGCG-SeNPs on hepatic and renal OS markers in 5-FU-provoked hepato-renal toxicity. Hepatic levels of (**A**) MDA, (**B**) NGAL, (**C**) GSH, (**D**) SOD, (**E**) CAT, (**F**) GR, (**G**) HO-1, and (**H**) Nrf2, and renal levels of (**I**) MDA, (**J**) NGAL, (**K**) GSH, (**L**) SOD, (**M**) CAT, (**N**) GR, (**O**) HO-1, and (**P**) Nrf2. Treatments markedly attenuated 5-FU–induced oxidative damage and restored antioxidant and cytoprotective signaling. Data are expressed as mean ± SEM (*n* = 7). Statistical significance among groups is indicated by asterisks; *: *p* < 0.05, **: *p* < 0.005, ***: *p* < 0.0005, ****: *p* < 0.00005, ns: non-significant.

As illustrated in Figure 6, administration of 5-fluorouracil (5-FU) induced profound and parallel oxidative stress in both hepatic and renal tissues, as evidenced by marked disturbances in lipid peroxidation, tubular injury markers, antioxidant defenses, and cytoprotective signaling pathways. Compared to the normal control group, 5-FU-treated rats exhibited pronounced elevations in malondialdehyde (MDA) in hepatic and renal tissues 153.33% and 342.00%, respectively, accompanied by significant increases in neutrophil gelatinase-associated lipocalin NGAL by 100% in the liver and 90.50% in the kidney. These changes were associated with substantial reductions in reduced glutathione (GSH), superoxide dismutase (SOD), catalase (CAT), and glutathione reductase (GR) in hepatic tissue by 69.01%, 51.76%, 54.84%, and 68.09%, respectively and in renal tissue by 63.90%, 46.70%, −48.70%, and 51.30%, respectively. In parallel, the expression of heme oxygenase-1 (HO-1) and nuclear factor erythroid 2-related factor 2 (Nrf2) was markedly suppressed in the liver 58.51% and 62.86% and in the kidney 46.80% and −64.60%, confirming severe disruption of redox homeostasis and endogenous cytoprotective signaling in both organs following 5-FU intoxication (*p* < 0.05).

Co-treatment with sodium selenite (5FU + Na_2_SeO_3_) resulted in partial attenuation of oxidative and stress-related alterations in both tissues. Relative to the normal group, hepatic and renal MDA levels remained elevated by 13.76% and 52.20%, respectively, while NGAL levels persisted above control values by 9.38% in the liver and 57.60% in the kidney. Antioxidant defenses also remained compromised, with hepatic GSH, SOD, CAT, and GR reduced by 34.69%, 29.57%, 31.78%, and 32.09%, respectively, and renal levels reduced by 4.80%, 10.00%, 6.60%, and 10.30%, respectively. Similarly, HO-1 and Nrf2 expression in both organs remained below physiological levels, indicating incomplete restoration of antioxidant signaling pathways and limited efficacy of inorganic selenium in mitigating 5-FU-induced hepato-renal oxidative injury (*p* < 0.05).

In contrast, EGCG supplementation (5FU + EGCG) substantially alleviated oxidative stress and improved antioxidant capacity in both hepatic and renal tissues. Compared to the 5-FU group, MDA levels declined in the liver and kidneys by 38.44% and 74.40%, respectively, while NGAL levels decreased by 38.44% in hepatic tissue and 42.10% in renal tissue. Concurrently, GSH, SOD, CAT, and GR activities increased in the liver by 41.53%, 44.35%, 39.92%, and 48.84%, respectively and in the kidney by 112.50%, 87.50%, 95.90%, and 106.00%, respectively. In addition, HO-1 and Nrf2 expression was markedly upregulated in both organs, reflecting partial reactivation of endogenous antioxidant and cytoprotective pathways; however, several parameters remained incompletely normalized relative to control values (*p* < 0.05).

Notably, treatment with FU + EGCG-SeNPs produced the most pronounced and coordinated restoration of oxidative balance and stress response signaling in both hepatic and renal tissues. Relative to the 5-FU group, hepatic and renal MDA levels declined by 61.84% and 82.50%, respectively, while NGAL levels decreased by 43.08% in the liver and 46.30% in the kidney. In parallel, GSH, SOD, CAT, and GR activities increased markedly in hepatic tissue by 213.64%, 90.24%, 82.14%, and 220.00%, respectively and in renal tissue by 176.80%, 122.20%, 128.20%, and 169.20%, respectively.

#### 2.2.4. Inflammatory Markers and Gene Expression in Hepatic and Renal Tissue

**Figure 7:**
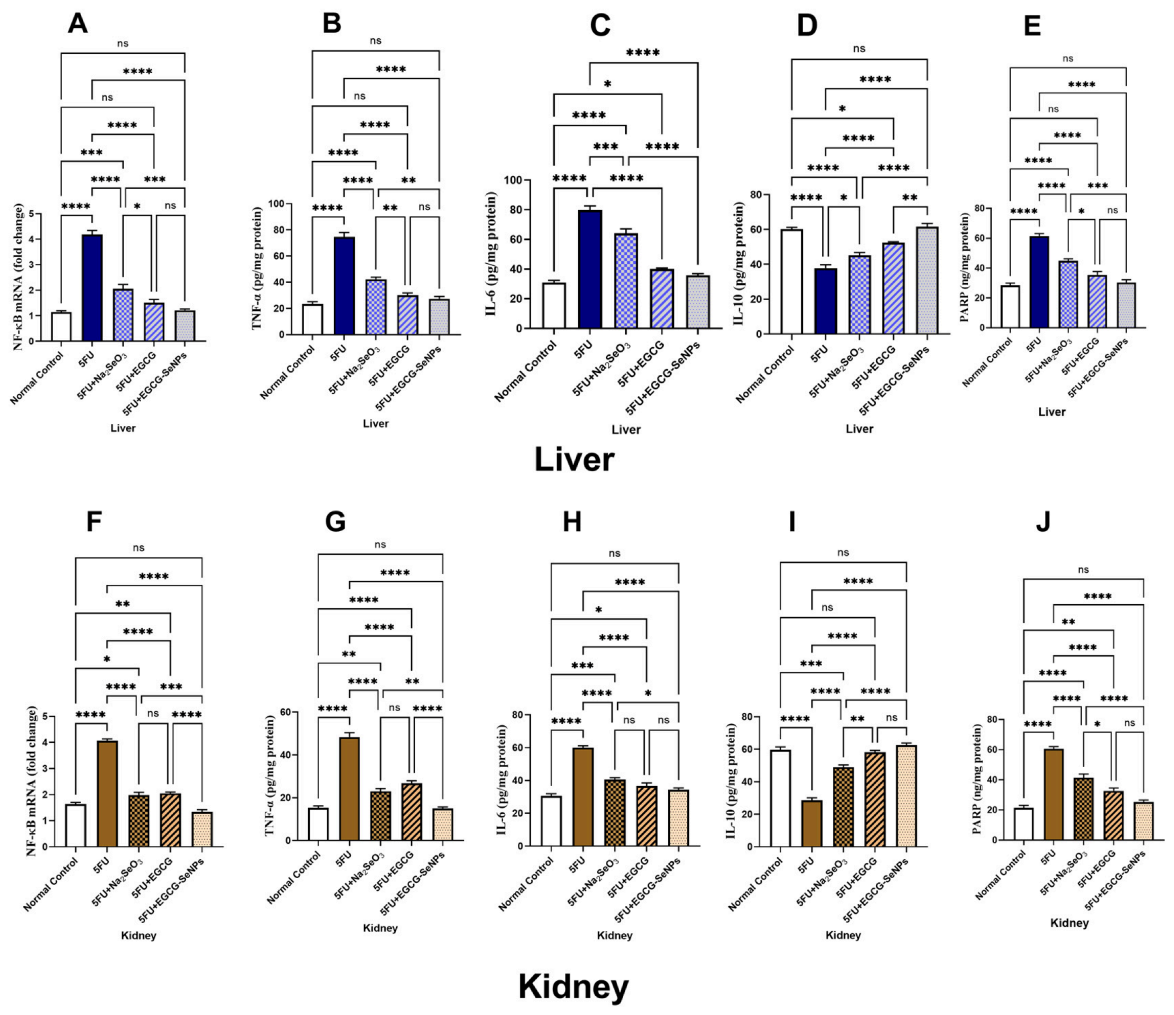
Impact of Na_2_SeO_3_, EGCG, and EGCG-SeNPs on hepatic and renal inflammatory markers and *NF-κB* gene expression in 5-FU–induced hepato-renal toxicity. Hepatic levels of (**A**) *NF-κB* mRNA expression, (**B**) TNF-α, (**C**) IL-6, (**D**) IL-10, and (**E**) PARP, and renal levels of (**F**) *NF-κB* mRNA expression, (**G**) TNF-α, (**H**) IL-6, (**I**) IL-10, and (**J**) PARP. Treatments attenuated 5-FU–induced inflammatory responses and normalized cytokine imbalance. Data are expressed as mean ± SEM (*n* = 7). Statistical significance among groups is indicated by asterisks; *: *p* < 0.05, **: *p* < 0.005, ***: *p* < 0.0005, ****: *p* < 0.00005, ns: non-significant.

As shown in Figure 7, 5-fluorouracil (5-FU) elicited a pronounced inflammatory response in both hepatic and renal tissues, characterized by marked upregulation of pro-inflammatory cytokines and activation of nuclear factor kappa-light-chain-enhancer of activated B cells (*NF-κB*) signaling. Compared with the normal control group, Tumor necrosis factor-alpha (TNF-α) and interleukin-6 (IL-6) levels were markedly increased in the liver 313.68% and 362.89%, respectively and in the kidney by 127.59% and 100%, respectively, accompanied by significant upregulation of *NF-κB* gene expression in hepatic by 282.69% and renal by 96.77% tissues. These alterations were associated with suppression of IL-10 in the liver 58.65% and dysregulated elevation in the kidney 61.76%. In parallel, poly ADP-ribose polymerase (PARP) protein levels were significantly increased in hepatic tissue by 248.15% and renal tissue by 90.91%, indicating enhanced inflammatory-associated cellular stress following 5-FU intoxication (*p* < 0.05).

Sodium selenite co-treatment (5FU + Na_2_SeO_3_) resulted in only partial attenuation of inflammatory disturbances. Pro-inflammatory cytokines, *NF-κB* expression, and PARP levels remained significantly elevated in both organs relative to the normal control values, whereas IL-10 remained suppressed in hepatic tissue and was abnormally regulated in renal tissue, reflecting limited anti-inflammatory efficacy.

In contrast, EGCG supplementation (5FU + EGCG) significantly mitigated inflammatory signaling in both tissues. Compared to the 5-FU group, TNF-α and IL-6 levels declined in hepatic by 56.40%, 55.46% and renal tissues, accompanied by significant downregulation of *NF-κB* and reduction of PARP protein levels. IL-10 levels were markedly increased in hepatic tissue 103.10% with partial normalization in renal tissue; however, inflammatory markers remained incompletely restored compared to controls (*p* < 0.05).

Notably, 5FU + EGCG-SeNPs produced the most pronounced and coordinated suppression of inflammation in both organs. Compared to the 5-FU group, TNF-α and IL-6 levels were markedly decreased in the liver by 71.69%, and 71.71% and kidney by 50.00%, and 45.31%, along with robust suppression of *NF-κB* expression and PARP protein levels. Concurrently, IL-10 levels were significantly upregulated in both tissues, approaching near-physiological levels. Relative to the normal control group, inflammatory markers exhibited only minimal deviations, and inflammatory markers were markedly suppressed, exhibiting remarkable improvement compared to the 5-FU group and approaching control levels (*p* < 0.05).

#### 2.2.5. Apoptotic Markers in Hepatic and Renal Tissues 

**Figure 8:**
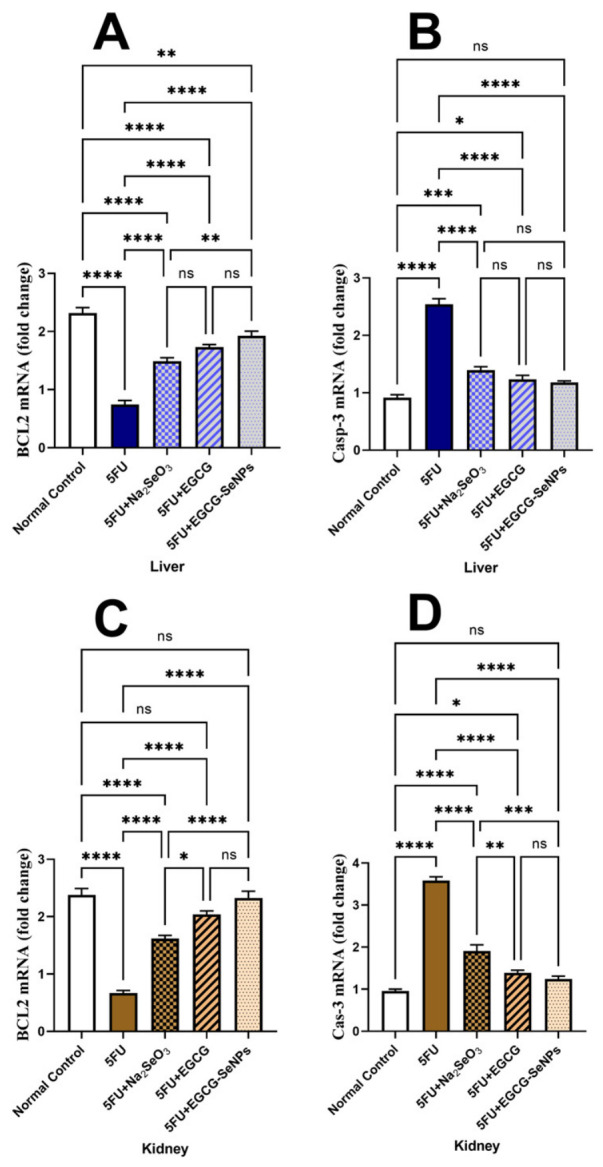
Effect of treatments on apoptotic gene expression in hepatic and renal tissues in 5-FU–induced hepato-renal toxicity. Relative mRNA expression of (**A**) *BCL-2* and (**B**) *caspase-3* in liver and (**C**) *BCL-2* and (**D**) *caspase-3* in kidney, reflecting apoptotic activity. Na_2_SeO_3_, EGCG, and EGCG-SeNPs treatments modulated apoptotic signaling induced by 5-FU. Data are expressed as mean ± SEM (*n* = 7). Statistical significance among groups is indicated by asterisks; *: *p* < 0.05, **: *p* < 0.005, ***: *p* < 0.0005, ****: *p* < 0.00005, ns: non-significant.

As shown in Figure 8, 5-fluorouracil (5-FU) induced pronounced apoptotic activation in both hepatic and renal tissues. Compared to the normal control group, 5-FU-treated rats exhibited significant downregulation of B-cell lymphoma 2 (*BCL-2*) in the liver and kidneys 58.82% and 61.54%, respectively accompanied by marked upregulation of *caspase-3* (233.33% and 250.00%, respectively; *p* < 0.05), indicating activation of mitochondrial-dependent apoptotic pathways in both organs.

Co-treatment with sodium selenite (5FU + Na_2_SeO_3_) resulted in modest modulation of apoptotic signaling, with *BCL-2* remaining significantly reduced in hepatic and renal tissues by 35.29% and 38.46%, and *caspase-3* remaining elevated by 100% and 111.11%, respectively (*p* < 0.05). These changes were not significant when compared with the 5-FU group, reflecting the limited anti-apoptotic efficacy of inorganic selenium.

In contrast, EGCG treatment (5FU + EGCG) significantly attenuated apoptosis in both organs, as evidenced by upregulation of *BCL-2* by 42.86% and 45.45% and downregulation of *caspase-3* by 46.67% and 48.00% in hepatic and renal tissues, respectively (*p* < 0.05). However, apoptotic markers remained significantly different from those in the normal control, indicating partial recovery.

Notably, EGCG mediated selenium nanoparticles (5FU + EGCG-SeNPs) exhibited the highest anti-apoptotic potential. Compared with the 5-FU group, *BCL-2* expression increased significantly 71.43% in the liver and 75.00% in the kidney, while *caspase-3* expression decreased by 66.67% and 68.00%, respectively; *p* < 0.05. In comparison with the normal control, apoptotic markers exhibited minimal deviations, and values were significantly improved compared to all other treatment groups.

#### 2.2.6. Histopathological Analysis

**Figure 9:**
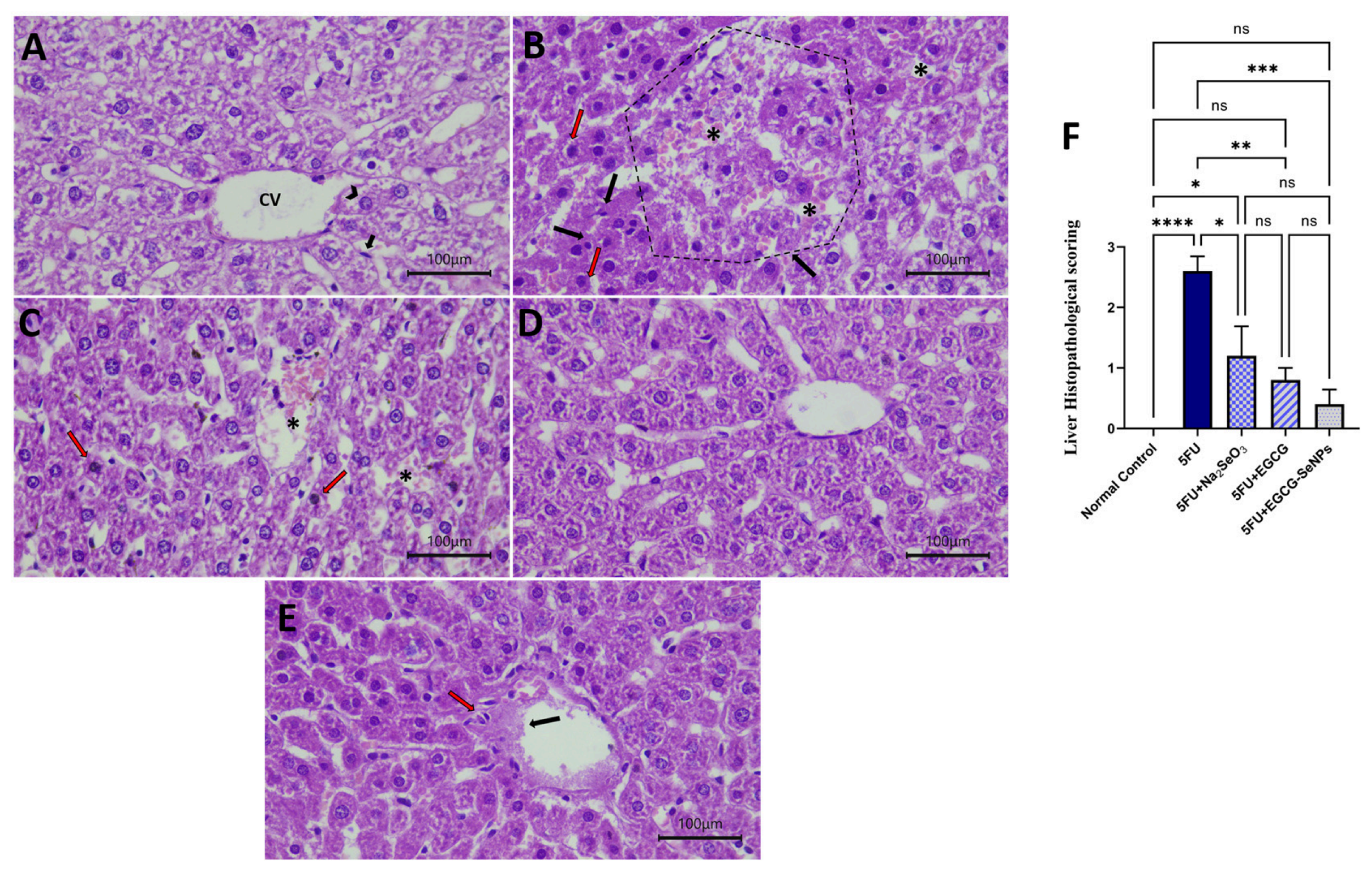
Histopathological examination of all groups. (**A**) The control group demonstrated typical hepatic structure, with well-organized hepatocyte cords emanating from the central vein (CV), including Kupffer cells (short arrow), intact cytoplasm, and clearly defined nuclei (arrow head). In contrast, (**B**) The 5FU group showed significant liver damage marked by widespread cytoplasmic degeneration and localized necrosis (dash-lined) with pyknotic cells (black arrow) accompanied by interlobular hemorrhage (*) and hyperchromatic hepatocytes (red arrow). (**C**) The 5FU + Na_2_SeO_3_ group demonstrated apparently normal hepatocytes in the centrilobular area, with congested central vein with extravasation and few hyperchromatic hepatocytes. (**D**) The 5FU + EGCG and (**E**) 5FU + EGCG-SeNPs groups revealed a substantial restoration of hepatic structure, displaying nearly typical hepatocytes, with recovery of cellular integrity. (**E**) 5FU + EGCG-SeNPs showed minimal inflammatory cell infiltration and hyaline material inside the central vein (H&E, ×400). (**F**) Semi-quantitative histopathological analysis of all experimental groups was expressed as mean ± S.E.M (*n* = 5). Statistical analysis by one-way ANOVA with Tukey’s post hoc test. Statistical significance among groups is indicated by asterisks; *: *p* < 0.05, **: *p* < 0.005, ***: *p* < 0.0005, ****: *p* < 0.00005, ns: non-significant.

**Figure 10:**
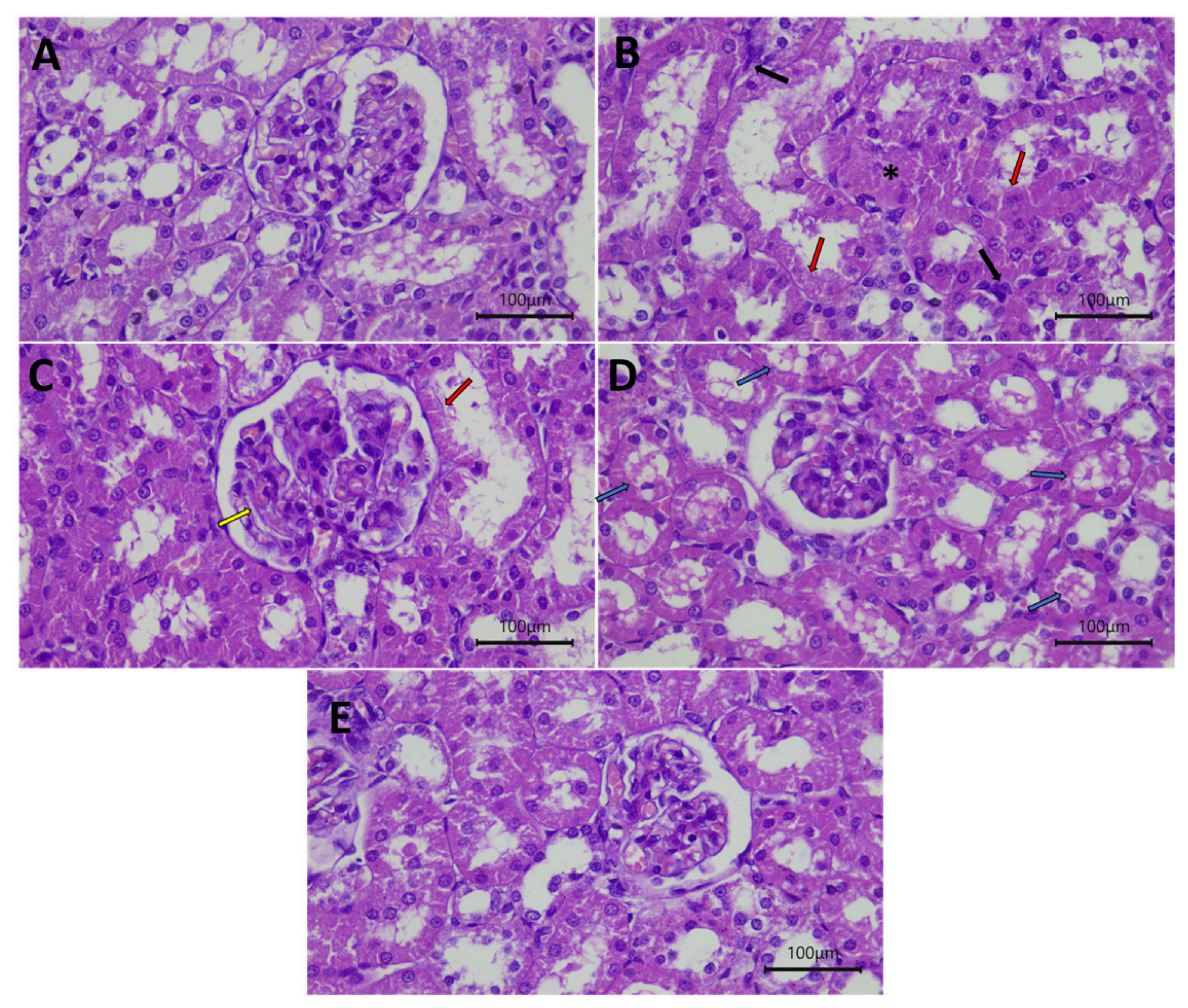
Photomicrographs of renal cortical tissues of all groups. (**A**) The control group showed normal glomeruli and convoluted tubules of the renal cortex. In contrast, (**B**) the 5FU group exhibited focal necrosis (*) with pyknotic cells (black arrow) accompanied by interstitial edema and necrotic cuboidal cells (red arrow). (**C**) The 5FU + Na_2_SeO_3_ group showed congested glomerular capillary tuft (yellow arrow) and minimal necrotic tubular cells (red arrow). (**D**) 5FU + EGCG group showed cytoplasmic vacuolation in the tubular epithelial cells (blue arrow). (**C**) The 5FU + Na_2_SeO_3_, (**D**) 5FU + EGCG, and (**E**) 5FU + EGCG-SeNPs groups showed markedly improved cortical structure, and (**E**) 5FU + EGCG-SeNPs group demonstrating an almost normal renal cortex structure and intact tubules. (H&E, ×400).

Control hepatic tissues exhibited a normal histological structure of hepatic lobules with vesicular hepatocytic nuclei radiating from the central vein. The 5FU treatment group displayed a remarkable pathological pattern of focal necrotic areas with pyknotic cells, hyperchromatic nuclei, and interlobular hemorrhage and loss of arrangement. The sodium selenite-treated group showed extravasated red blood cells from the central vein and a few hyperchromatic hepatocytes. Both the EGCG- and EGCG-SeNPs-treated groups revealed an approximately normal histological architecture of hepatic tissue (Figure 9). The renal tissues of control group revealed normal structure of renal cortex with glomeruli, proximal and distal convoluted tubules. The other treated groups showed normal cuboidal cells of the proximal convoluted tubules and intact glomeruli, indicating the potential protective effects of Na_2_SeO_3_, EGCG, and EGCG-SeNPs against the harmful effects of 5FU (Figure 10). The other treated groups showed normal cuboidal cells of the proximal convoluted tubules and intact glomeruli giving evidence of the potential effect of Na_2_SeO_3_, EGCG, and EGCG-SeNPs in protecting renal tissues from the harmful impact of 5FU (Figure 10, Table 1).

#### 2.2.7. IHC Analysis

**Figure 11:**
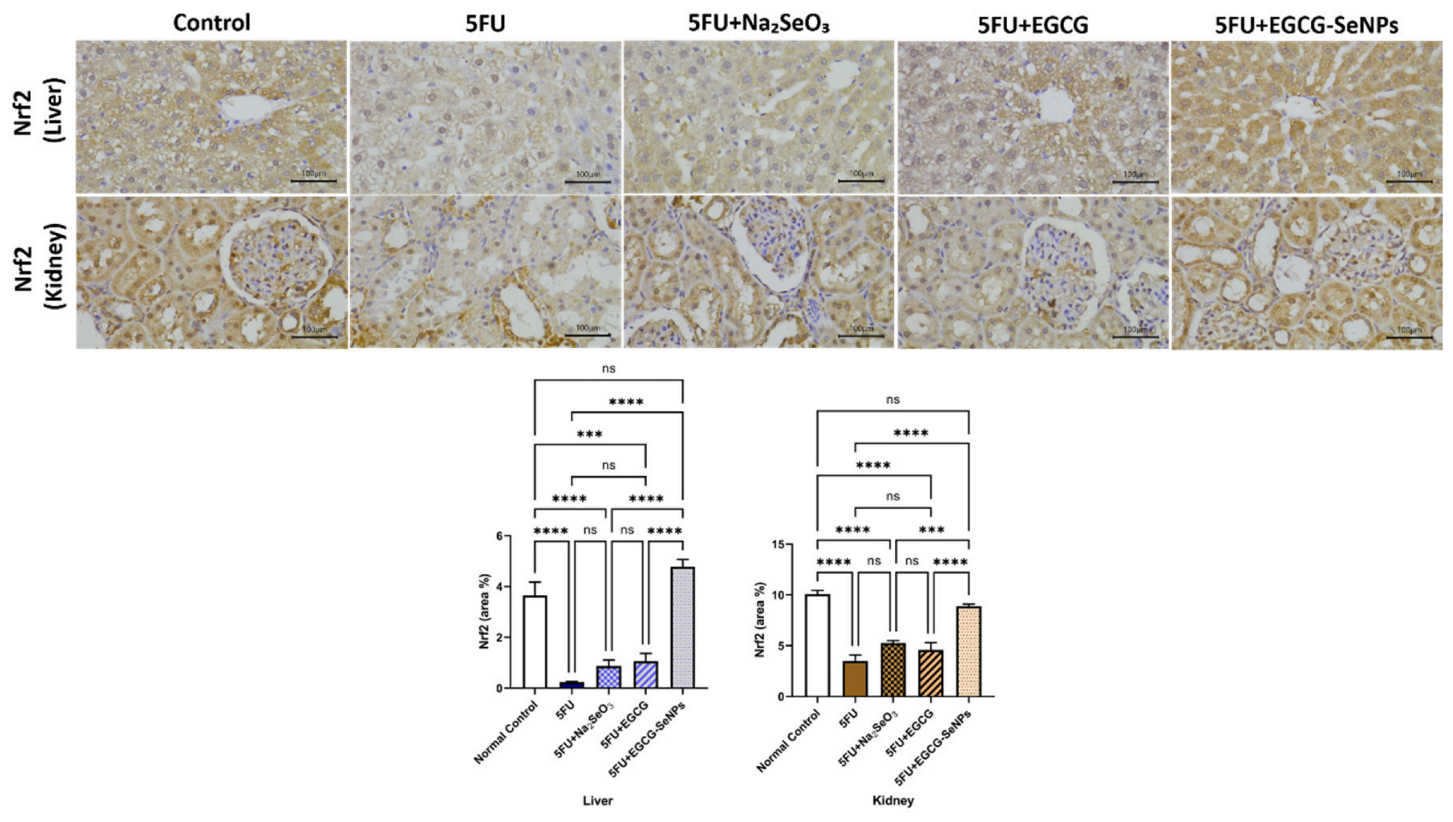
Impact of EGCG-SeNPs on hepatic and renal Nrf2 reactivity. Nrf-2 reactivity was apparent in the cells as a brown color (arrow) (DAB, ×400). Quantitative analysis of IHC for Nrf-2 is expressed as the mean ± SEM (*n* = 7). Statistical analysis by one-way ANOVA with Tukey’s post hoc test. Statistical significance among groups is indicated by asterisks; ***: *p* < 0.0005, ****: *p* < 0.00005, ns: non-significant.

IHC analysis showed marked Nrf-2 expression in hepatic and renal tissues of the control and 5FU+ EGCG-SeNPs-treated groups, whereas 5FU + Na_2_SeO_3_ and 5FU + EGCG groups revealed moderate expression. 5FU showed weak expression of Nrf-2. There was remarkable variation between the 5-FU and 5-FU +EGCG-SeNPs groups regarding Nrf-2 IHC immunoreactivity (*p* < 0.001), with substantially increased Nrf-2 positivity in the 5-FU +EGCG-SeNPs group (Figure 11).

**Figure 12:**
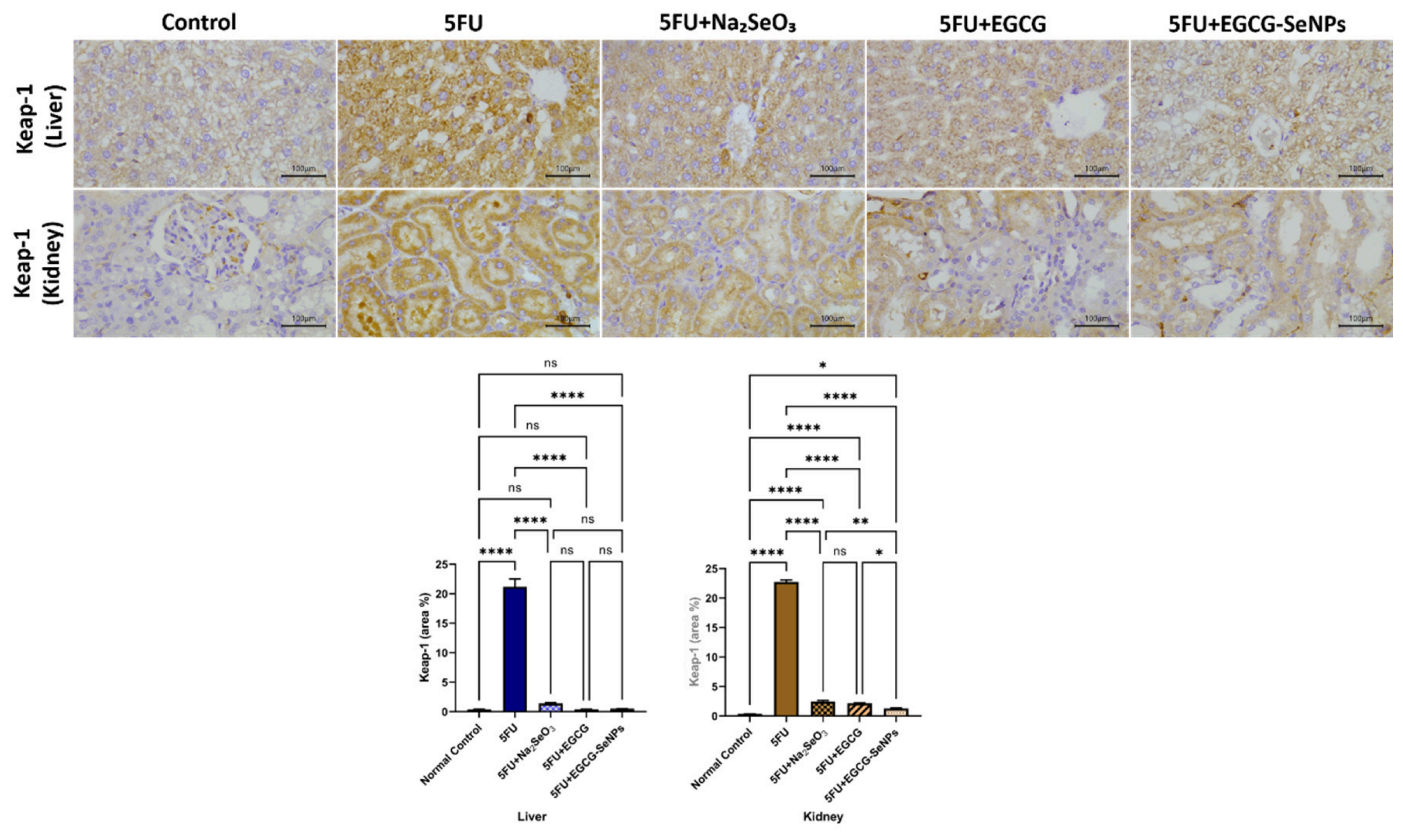
Impact of EGCG-SeNPs on hepatic and renal reactivity of Keap-1. Nrf-2 reactivity was apparent in the cells as a brown color (arrow) (DAB, ×400). Quantitative analysis of IHC for Keap-1 is expressed as the mean ± SEM (*n* = 7). Statistical analysis by one-way ANOVA with Tukey’s post hoc test. Statistical significance among groups is indicated by asterisks; *: *p* < 0.05, **: *p* < 0.005, ****: *p* < 0.00005, ns: non-significant.

Moreover, Keap-1 was weakly expressed in hepatic and renal tissues of the control, Na_2_SeO_3_, EGCG-5FU, and EGCG-SeNPs-5FU groups. However, the 5FU group showed marked Keap-1 expression. There was a remarkable difference between the 5FU and EGCG-SeNPs-5FU groups in terms of Keap-1 IHC immunoreactivity (*p* < 0.001), with a substantial decline in Keap-1 positivity in the EGCG-SeNPs-5FU group compared to the 5FU group (Figure 12).

## 3. Discussion

The EGCG-functionalized of SeNPs was verified through physicochemical analysis, revealing stable nanostructures with enhanced characteristics. These nanoparticles showed consistent nanoscale distribution, spherical shapes, and negative surface charge, indicating successful reduction by EGCG [7]. Moreover, physicochemical attributes determine nanoparticle behavior in biological systems, as nanoscale dimensions enhance the surface-to-volume ratio and reactivity. The negative surface charge prevents aggregation and ensures dispersion, maintaining bioavailability [11]. The structural characteristics are associated with the increased antioxidant capacity of SeNPs synthesized with EGCG. The smaller size allows for improved access within cells and more effective scavenging of reactive oxygen species, whereas EGCG attached to the surface offers additional antioxidant benefits [7,12]. The synergistic integration of selenium’s redox-modulating properties with the polyphenolic antioxidant capacity of EGCG likely enhances the nanoparticles’ ability to restore redox homeostasis and protect against oxidative damage. Additionally, improved colloidal stability and cellular interactions increase the likelihood of effectively modulating redox-sensitive signaling pathways, such as those governing antioxidant defense and inflammatory responses [7].

Chemotherapy-associated toxicity remains a major obstacle in cancer management and often requires dose reduction. 5-Fluorouracil (5-FU) is effective against solid tumors but causes substantial off-target toxicity in metabolically active organs, particularly the liver and kidneys [13,14]. This study showed that 5-FU induces hepato-renal injury through oxidative stress (OS), inflammation, and apoptosis. Our results demonstrate that EGCG-SeNPs provide superior protection against these processes compared with EGCG or inorganic selenium alone.

The liver and kidneys are inherently susceptible to 5-FU-induced toxicity because of their central roles in drug metabolism, detoxification, and excretion. Hepatocytes are directly exposed to reactive intermediates generated during the hepatic biotransformation of 5-FU [15], whereas renal tissues, particularly proximal tubular epithelial cells, are continuously exposed to circulating xenobiotics and oxidative metabolites. Recent studies have emphasized that injury in these organs is not merely a direct toxic effect of chemotherapeutic agents but rather a consequence of dysregulated cellular stress responses that evolve into sustained tissue damage [12,16].

Oxidative stress is widely regarded as the initiating trigger of 5-FU-induced hepatorenal toxicity. Recent studies have demonstrated that metabolic activation of 5-FU leads to overproduction of reactive oxygen species (ROS), overwhelming endogenous antioxidant systems, and disrupting cellular redox balance [15,17], The marked elevation in MDA following 5-FU administration indicates severe lipid peroxidation due to excessive ROS generation. The greater increase in renal tissue may reflect the high metabolic activity and mitochondrial density of renal tubular cells, which make them especially vulnerable to oxidative damage. In hepatic tissue, this redox imbalance promotes lipid peroxidation, protein oxidation, and mitochondrial dysfunction, resulting in compromised membrane integrity and impaired metabolic activity [18]. In the kidney, high mitochondrial density and oxygen consumption render tubular cells particularly vulnerable to oxidative injury, leading to disruption of tubular transport mechanisms and cellular degeneration [19]. Although NGAL is widely used as a biomarker of renal injury, it is not exclusively kidney specific. NGAL is an acute-phase protein that can also be expressed in hepatic tissue, particularly under conditions of oxidative stress and inflammation. Its expression is regulated by inflammatory signaling pathways, including NF-κB, and has been reported to increase in the liver in response to systemic toxic insults. Importantly, NGAL has been shown to serve as a prognostic biomarker in chronic liver diseases, supporting its functional relevance in hepatic pathology. Therefore, the observed elevation of NGAL in hepatic tissue in the present study reflects the inflammatory and oxidative stress status of the liver, supporting the systemic nature of 5-FU-induced toxicity [20]. The depletion of enzymatic and non-enzymatic antioxidants further exacerbates oxidative damage, establishing a persistent pro-oxidant state [21].

Mitochondria represent a critical nexus linking oxidative stress to downstream pathological events. Overproduction of reactive oxygen species (ROS) destabilizes mitochondrial membranes, impairs electron transport, and reduces ATP synthesis, thereby compromising cellular energy homeostasis [22]. Recent investigations have highlighted mitochondrial dysfunction as a central determinant of chemotherapy-induced organ injury, as it facilitates the release of pro-apoptotic mediators and sensitizes cells to death signals. Thus, mitochondrial impairment serves as a key mechanism by which oxidative stress transitions from reversible injury to irreversible cellular loss [23].

Inflammatory signaling constitutes the second major axis of 5-FU-induced toxicity and is closely intertwined with OS. Activation of redox-sensitive transcription factors, particularly NF-κB, may have a potential impact on this process. Reactive oxygen species act as potent activators of NF-κB, promoting the transcription of pro-inflammatory cytokines and chemokines [24]. In hepatic tissue, sustained NF-κB activation disrupts hepatocellular architecture, impairs synthetic and metabolic functions, and suppresses regenerative capacity. In renal tissue, NF-κB-driven inflammation contributes to endothelial dysfunction [25], tubular epithelial injury, and immune cell infiltration, thereby accelerating renal functional decline. Recent studies have emphasized that persistent NF-κB activation is a hallmark of chemotherapy-induced organ inflammation and a key contributor to chronic tissue damage [26].

A critical feature of 5-FU-induced injury is bidirectional amplification between oxidative stress and inflammation. Inflammatory cells recruited to damaged tissues generate additional reactive oxygen species, thereby intensifying oxidative injury, whereas oxidative stress continuously reinforces inflammatory transcriptional programs [27]. Concurrent suppression of anti-inflammatory mediators further impairs the resolution of inflammation, resulting in a self-perpetuating cycle of tissue damage. This sustained inflammatory environment primes cells for apoptotic signaling and limits the capacity for tissue repair [28].

Moreover, apoptosis represents the final execution phase of 5-FU-induced hepatorenal toxicity. Current evidence indicates that the convergence of OS and inflammatory signaling disrupts the eqilibium between pro- and anti-apoptotic regulators, favoring activation of intrinsic, mitochondria-dependent apoptotic pathways. In the liver, apoptotic loss of hepatocytes compromises detoxification and metabolic capacity and disrupts tissue architecture [29]. In the kidney, apoptosis of tubular epithelial cells contributes to tubular atrophy, impaired resorptive function, and the progression of nephrotoxicity. Importantly, apoptotic debris can further stimulate inflammatory responses, reinforcing the pathological loop linking OS, inflammation, and apoptosis [30].

Therefore, the limited protective efficacy observed with sodium selenite reflects the constraints of conventional antioxidant supplementation in the context of severe chemotherapeutic stress. Although Se is a vital component of glutathione peroxidase, inorganic selenium exhibits restricted bioavailability and a narrow therapeutic window. Recent studies have highlighted that under intense oxidative and inflammatory conditions, inorganic selenium alone is insufficient to fully restore redox balance or suppress inflammatory and apoptotic signaling, thereby limiting its protective potential against chemotherapy-induced organ toxicity [22]. EGCG supplementation provides more substantial protection by targeting multiple components of the injury cascade [31]. EGCG is a well-characterized polyphenol with potent antioxidant and anti-inflammatory properties. Recent research has shown that EGCG effectively scavenges reactive oxygen species, enhances endogenous antioxidant defenses, and inhibits NF-κB activation. Additionally, EGCG stabilizes cellular membranes and preserves mitochondrial integrity, thereby limiting apoptotic progression [27]. However, despite these multifaceted actions, EGCG alone does not fully normalize pathological alterations, likely due to limitations in bioavailability, rapid metabolism, and insufficient intracellular accumulation during severe toxic stress [32].

The most pronounced and comprehensive protection was achieved with EGCG mediated selenium nanoparticles [33]. Recent advances in nanomedicine have underscored the advantages of nanoparticle-based delivery systems in enhancing bioavailability, improving cellular uptake, and reducing systemic toxicity [34]. In the present study, EGCG-SeNPs effectively restored redox homeostasis by reducing oxidative burden and replenishing antioxidant defenses in both hepatic and renal tissues. Simultaneously, they exerted strong inhibitory effects on NF-κB signaling, suppressing downstream inflammatory cascades, and interrupting the feed-forward loop linking oxidative stress and inflammation [35].

At the apoptotic level, EGCG-SeNPs demonstrated superior efficacy in preserving mitochondrial integrity and re-establishing the balance between pro- and anti-apoptotic regulators. By limiting excessive caspase activation, EGCG-SeNPs reduced apoptotic cell loss and preserved tissue architecture [36]. Recent studies have suggested that the synergistic interaction between polyphenols and selenium at the nanoscale enhances the modulation of stress-responsive signaling pathways, including NF-κB and Nrf2, thereby providing integrated cytoprotection [37].

Notably, the parallel protective effects observed in both the hepatic and renal tissues highlight the systemic efficacy of EGCG-SeNPs and underscore the functional interdependence of these organs. The contemporary literature increasingly recognizes hepatorenally crosstalk as a critical determinant of multi-organ toxicity during chemotherapy. EGCG-SeNPs offer a distinct therapeutic advantage in mitigating chemotherapy-associated systemic toxicity by protecting both organs simultaneously [37].

Although EGCG-SeNPs demonstrated marked hepatorenally protective effects against 5-FU-induced toxicity, the potential for drug–drug interactions must be carefully considered when proposing any adjunctive therapy during chemotherapy. Notably, 5-fluorouracil is predominantly metabolized by dihydropyrimidine dehydrogenase rather than cytochrome P450-dependent pathways, reducing the likelihood that EGCG-mediated enzyme modulation would significantly alter its systemic clearance. In the present study, the protective effects of EGCG-SeNPs were primarily associated with the attenuation of oxidative stress, activation of the Nrf2/HO-1 cytoprotective axis, suppression of NF-κB-driven inflammatory signaling, and inhibition of mitochondrial-dependent apoptosis in hepatic and renal tissues, rather than indications of altered drug metabolism. Moreover, nano-functionalization of EGCG onto selenium nanoparticles enables controlled delivery and limits excessive free EGCG exposure, which may otherwise contribute to pharmacokinetic interference. Importantly, EGCG-SeNPs restored biochemical, molecular, and histopathological parameters to physiological levels without inducing abnormal changes in control animals, supporting a cytoprotective rather than a pharmacologically antagonistic role. Nevertheless, the current study focused on toxicity mitigation and did not directly assess tumor response or 5-FU pharmacokinetics; therefore, future investigations incorporating tumor-bearing models and metabolic profiling are warranted to conclusively exclude any impact on chemotherapeutic efficacy.

This study underscores the protective benefits of EGCG-SeNPs; however, it may fall short in providing detailed mechanistic insights into the specific pathways responsible for these effects. Further research is necessary to delve into the molecular mechanisms underlying the observed outcomes. Moreover, the study did not include a long-term follow-up to assess the potential delayed effects of 5-FU and the sustained efficacy of EGCG-SeNPs. Evaluating long-term results would offer valuable insights into the safety and effectiveness of treatment. Additionally, the study did not explore possible interactions between EGCG-SeNPs and other treatments that patients might receive during chemotherapy. Understanding these interactions is essential for developing effective combination therapies. Future studies will quantify EGCG loading using appropriate analytical methods such as HPLC or UV–Vis analysis. A limitation of the present study is the lack of Western blot analysis to provide quantitative protein-level validation of the proposed modulation of Nrf2/HO-1 and NF-κB signaling pathways, as well as the exact EGCG loading on the surface of EGCG-SeNPs was not quantified. Addressing these limitations in future studies will enhance the understanding of the therapeutic potential of EGCG-SeNPs and their applicability in clinical settings.

## 4. Materials and Methods

### 4.1. Chemical Synthesis of EGCG-Functionalized Selenium Nanoparticles

Epigallocatechin-3-gallate (EGCG; CAS No. 989-51-5, analytical grade) was purchased from Sigma-Aldrich (St. Louis, MO, USA). Sodium selenite (Na_2_SeO_3_; Cat. No. 214485-5G, ≥98% purity) was obtained from Sigma-Aldrich (St. Louis, MO, USA). Bioreductive formulation of EGCG mediated SeNPs (EGCG-SeNPs) was achieved via a bioreductive method, wherein equal aliquots (10 mL each) of sodium selenite (Na_2_SeO_3_, 10 mM) and EGCG solution (3.5 mg/mL) were mixed under constant magnetic stirring at room temperature for approximately 12 h. The resulting colloidal suspension was dried to obtain a fine, stable EGCG-SeNPs powder, which was then preserved for detailed physicochemical characterization and biological experimentation. The gradual emergence of reddish coloration visually confirmed the successful formation of selenium nanoparticles. The resulting colloidal suspension was dried to obtain a fine, stable EGCG-SeNPs powder, which was then preserved for detailed physicochemical characterization and biological experimentation.

### 4.2. Physico-Chemical Characterization of Selenium Nanoparticles (EGCG-SeNPs)

The physicochemical properties of EGCG-SeNPs were characterized using a Zetasizer Nano ZS90 (Malvern Panalytical Ltd., Worcestershire, UK). Dynamic light scattering (DLS) revealed a narrow particle size distribution and high dispersion quality, whereas zeta potential analysis indicated a negatively charged surface, ensuring colloidal stability [38]. The samples were sonicated to prevent agglomeration. Additionally, transmission electron microscopy (TEM) images obtained at Al-Azhar University confirmed the uniform size, spherical morphology, and structural integrity of the nanoparticles in the colloidal matrix [39].

### 4.3. UV–Visible Spectroscopic Assessment

The optical characteristics and spectral measurements of EGCG-SeNPs were conducted using a Shimadzu UV–Vis. spectrophotometer (Shimadzu Corporation, Kyoto, Japan). Absorbance spectra were collected in the wavelength range of 200–800 nm to assess the optical response and identify characteristic absorption features associated with nanoparticle formation.

### 4.4. FT-IR and XRD Analysis

The synthesized nanoparticles were analyzed using an ATR-FTIR spectrophotometer in the Nano Center, Faculty of Engineering, Capital University. Under standard operating conditions, the dried nanoparticle samples were directly placed on a diamond ATR crystal, and spectra were obtained in the wavenumber range of 4000–400 cm^−1^.

X-ray diffraction (XRD) analysis was performed to determine the structural characteristics of the synthesized EGCG-functionalized selenium nanoparticles (EGCG-SeNPs). The diffraction pattern was recorded using an X-ray diffractometer under standard operating conditions over a scanning range of 10–80° (2θ) at the Faculty of Engineering, Capital University, Egypt. The analysis was conducted to assess the crystallinity and phase structure of the synthesized nanoparticles.

### 4.5. Biological Experimental Design

Thirty-five healthy adults male Wistar albino rats, with body weights ranging between 120 and 150 g, were obtained from the Vacsera Animal House, Helwan, Cairo, Egypt. Upon arrival, the animals were housed two or three per cage and were housed in well-ventilated wire-mesh polypropylene cages and maintained under carefully controlled laboratory conditions. Environmental parameters were standardized to include a 12 h alternating light and dark photoperiod and an ambient temperature regulated at 25 ± 2 °C. Throughout the experimental period, the animals had free access to a commercially available standard rodent pellet diet and potable water. Prior to the commencement of experimental procedures, all rats were allowed an acclimatization period of two weeks to adapt to the housing conditions and minimize stress-related variability.

Thirty-five adult rats were arbitrarily assigned to five experimental groups (*n* = 7 per group) to investigate the potential hepatorenal-protective effects of selenium, epigallocatechin gallate (EGCG), and EGCG–SeNPs against 5-fluorouracil (5FU)-induced toxicity

Control group (normal control): rats in this group received oral normal saline (0.9% NaCl) throughout the experimental period and intraperitoneal (i.p.) saline injections corresponding to the 5-FU administration schedule.

5-fluorouracil (5-FU) group: rats in this group were administered 5-fluorouracil intraperitoneally at a dose of 30 mg/kg/day for five consecutive days (days 17–21) to induce hepatorenal injury, following previously established protocols [40,41].

5FU + Na_2_SeO_3_ group: rats in this group received sodium selenite (0.5 mg/kg/day, orally) once daily for 21 consecutive days [42,43] along with 5-FU administration as described above.

5FU + EGCG group: rats in this group received EGCG (100 mg/kg/day, orally) once daily for 21 consecutive days [44,45], along with 5-FU administration as described above.

5FU + EGCG group: rats of this group were treated with EGCG (100 mg/kg/day, orally) for 21 days, hepatorenal toxicity was induced by intraperitoneal injection of 5-fluorouracil (5-FU; 30 mg/kg/day) during the final five consecutive days (days 17–21), following established protocols [40,41].

5FU + EGCG-SeNPs group: rats in this group received EGCG-functionalized selenium nanoparticles (0.5 mg/kg/day, orally) once daily for 21 consecutive days [46], along with 5-FU administration as described above.

**Scheme 1:**
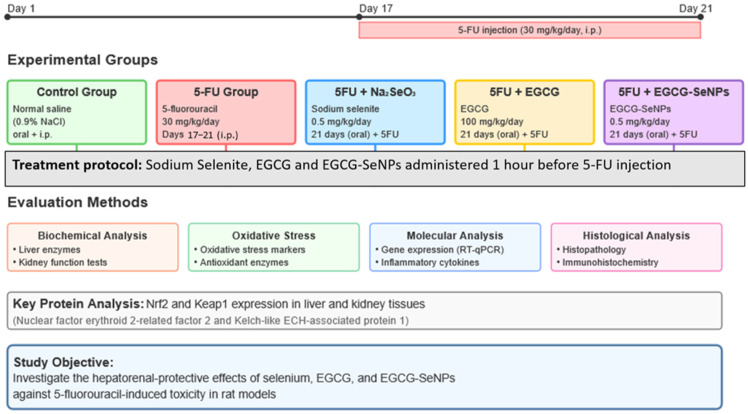
Hepatorenal-protective study design.

In all treated groups, EGCG and its nanoform were administered one hour prior to 5-FU injection to ensure optimal systemic availability and protective efficacy, all administered compounds were freshly prepared in distilled water prior to dosing to ensure proper dissolution and uniform oral delivery (Scheme 1).

### 4.6. Sample Collection and Tissue Preparation

Animals were monitored for clinical or behavioral signs of systemic toxicity during the experimental period. After completing treatment, rats were euthanized 24 h post-final dose to prevent acute pharmacodynamic interference. Blood samples were drawn via cardiac puncture, and serum was collected for hepatic and renal biochemical marker analysis. The liver and kidney tissues were excised and divided into three sections. The first section was weighed and homogenized in the same buffer to produce a 10% (*w*/*v*) tissue homogenate, centrifuged at 3000× *g* for 10 min at 4 °C, and the supernatant was collected for biochemical and oxidative stress analyses. The second section was frozen and stored at −80 °C for molecular and enzymatic studies, whereas the third section was fixed in 10% neutral-buffered formalin for histopathological examination. Prior to the study, all procedures complied with the ARRIVE guidelines and were approved by the Research Ethics Committee of Sinai University (approval no. SU.REC.2025 (86A)).

### 4.7. Serum Liver Enzymes Level, and Kidney Function Test Level

Serum ALT, AST, and ALP activities were quantified using standard colorimetric methods to assess hepatic function. ALT and AST were measured using (Cat. NO. AT 1034, and AS 1061, respectively) were purchased from bio-diagnostic at (Giza, Egypt), following [47], based on transamination reactions forming pyruvate or oxaloacetate, which were detected using 2,4-dinitrophenylhydrazine at 505 nm. ALP activity was determined using a Quimica Clinica Aplicada kit (Cat. No. 996265, Tarragona, Spain), according to Babson et al. [48], where enzymatic hydrolysis of phenolphthalein monophosphate releases a pink chromophore measurable at 510 nm.

Renal activity was evaluated by measuring serum creatinine and urea concentrations using commercial kits (Cat. Nos. CR 1250, and UR 2110, respectively) were purchased from bio-diagnostic kit (Giza, Egypt) in accordance with the standard protocol of Gouden et al. [49]. The assays rely on colorimetric detection at 505 nm, and concentrations were quantified through calibration with standard reference curves, providing an accurate estimation of renal functional integrity.

### 4.8. Evaluation of Hepato-Renal Oxidative Stress and Antioxidant Enzyme Activities

Oxidative stress biomarkers included lipid peroxidation (LPO) estimated as MDA following Ohkawa [50], and GSH concentration, which was determined by Ellman [51], while antioxidant defense enzymes SOD and CAT [52,53,54].

### 4.9. Determination of Inflammatory Cytokines in Hepato-Renal Tissues

Inflammatory cytokines, TNF-α, IL-6, and IL-10, were determined in hepatic and renal tissue homogenates using ELISA kits (MyBioSource, San Diego, CA, USA; Cat. Nos. MBS175904, MBS2702038, and MBS269138, respectively). All measurements were conducted in accordance with the manufacturer’s instructions to ensure precise detection of cytokine levels, which reflected tissue inflammatory responses.

### 4.10. Gene Expression Analysis Using Rt-qPCR

Total RNA was extracted from hepatic and renal tissues via TRIzol reagent (Cat. No. 15596026, Thermo Fisher Scientific, Carlsbad, CA, USA) following the manufacturer’s instructions, and cDNA was synthesized using the MultiScribe™ Reverse Transcriptase Kit (Cat. No. 4311235, Applied Biosystems, Foster City, CA, USA) Quantitative real-time PCR was conducted with Power SYBR™ Green Master Mix (Cat. No. 4309155, Applied Biosystems, Foster City, CA, USA) on an Applied Biosystems 7500 System to quantify Bcl-2, NF-kB and Caspase-3 gene expression (Table 2). Transcript values were normalized to β-actin and determined via the 2^–ΔΔCt^ method to assess apoptosis-related gene modulation. The threshold cycle numbers (Ct) of gene targets were standardized to the reference genes for both the experimental and control groups in accordance with the following equations [17]:

(1) ∆ Ct (tested = Ct (target in the teste groups) − Ct (ref. in test group)

(2) ∆ Ct (calibrator) = Ct (target in control) − Ct (ref. in control)

(3) ∆∆ Ct=C∆ Ct (test) − ∆ Ct (calibrator)

(4) $$Fold\;changes\;=\;2^{-∆∆Ct}$$

### 4.11. Histopathological Examination

Liver and kidney tissue specimens were carefully fixed, processed, and subjected to hematoxylin and eosin (H&E) staining to evaluate the overall histological structure. This included an analysis of nephron organization and identification of histopathological alterations within the tissues [55,56].

Hepatic damage scoring was rated as follows: 0—minimal or no injury; 1—mild damage with cytoplasmic vacuolization and focal nuclear pyknosis; 2—moderate injury with cytoplasmic vacuolization, without overt necrosis, along with sinusoidal dilation and congestion; 3—moderate injury, showing coagulative necrosis and extensive sinusoidal dilation/congestion; 4—severe injury with widespread coagulative necrosis and hemorrhage, resulting in tissue architecture loss [57,58].

Regarding histopathological scoring of renal tissues, the following features were evaluated; Vacuolation of tubular epithelium, Congestion and hypertrophy of glomerular tuft, Congestion of inter-tubular blood capillaries, and Focal tubular necrosis, these features were classified into negative (lack of histological alteration), mild change where <15% of the examined tissue sections is affected, moderate damage where 15–35% of the examined tissue sections is affected, and severe damage where >35% of the examined tissue sections is affected [59].

### 4.12. Immunohistochemical Analysis of Nrf2 and Keap1 in Liver and Kidney Tissues

Immunohistochemistry (IHC) was conducted on paraffin-embedded tissue sections (4–5 µm) from liver and kidney samples to evaluate the expression of Nrf2 (CAT # NBP3-13682; Novus Biologicals, Littleton, CO, USA) and its regulator Keap1 (CAT # NBP1-83106; Novus Biologicals, Littleton, CO, USA). The sections were initially deparaffinized, rehydrated through a graded series of alcohols, and subjected to antigen retrieval as necessary to unmask the epitopes. Following this, the tissue samples were incubated with primary antibodies specific to Nrf2 and Keap1. This was followed by incubation with species-specific biotinylated secondary antibodies and streptavidin–horseradish peroxidase (HRP) conjugates. The immune complexes were visualized via DAB. To counterstain the nuclei, sections were treated with hematoxylin. The stained tissue sections were subsequently examined under a light microscope to assess the localization and intensity of protein expression, providing insights into the cellular distribution and activation of Nrf2 signaling in the liver and kidney tissues [60,61].

### 4.13. Quantitative Assessment of IHC Staining

This procedure serves as a reliable approach for assessing protein localization within tissue architecture. Semi-quantitative analysis of immunohistochemical staining was performed using the ImageJ Fiji software (version 1.52n), with Hematoxylin and DAB (H-DAB) image deconvolution, and subsequent quantitative analysis was performed to evaluate staining intensity and distribution. The percentage area of Nrf-2 and Keap-1 immunoreactivity was quantified at a magnification of 400× in all experimental groups [62,63].

### 4.14. Statistical Analysis

Data are presented as the mean ± SEM. One-way ANOVA was used to compare groups, followed by Tukey’s post hoc test for multiple comparisons. All statistical analyses were performed using GraphPad Prism (version 9.0; GraphPad Software, San Diego, CA, USA). Statistical significance was set at *p* < 0.05. The percentage change relative to the control group was calculated using the following equation:

(5) $$\%\;of\;Difference\;=\;\frac{\mathrm{Treatrd}\;\mathrm{value}\;-\;\mathrm{control}\;\mathrm{value}}{\mathrm{Control}\;\mathrm{value}}\;\times \;100$$

## 5. Conclusions

In conclusion, the present study demonstrates that EGCG-functionalized selenium nanoparticles (EGCG-SeNPs) provide significant protection against 5-fluorouracil (5-FU)-induced hepatorenal toxicity. The dual action of EGCG as a reducing and stabilizing agent in the synthesis of selenium nanoparticles enhances their bioavailability and therapeutic efficacy. Our findings indicate that EGCG-SeNPs effectively restore liver and kidney function by modulating oxidative stress, inflammation, and apoptosis, primarily through the activation of the Nrf2/HO-1 signaling pathway and inhibition of NF-κB activation. This study underscores the potential of EGCG-SeNPs as a promising adjunctive therapy in chemotherapy, aiming to mitigate the adverse effects associated with 5-FU treatment. Future research should focus on elucidating the long-term safety and efficacy of EGCG-SeNPs in clinical settings and exploring their applications in other chemotherapeutic contexts to improve patient outcomes.

## Figures and Tables

**Table 1:** Renal histopathologic change in 5FU-provoked nephrotoxicity in rats.

Histopathological Change	Normal Control	5-FU	5FU + Na_2_SeO_3_	5-FU +EGCG	5-FU +EGCG-SeNPs
Vacuolation of tubular epithelium	−	+++	+	+	−
Congestion of inter-tubular blood capillaries	−	+	−	−	−
Congestion and hypertrophy of glomerular tuft	−	++	+	+	+
Focal tubular necrosis	−	+++	+	−	−

(+++) describes severe damage; (++) describes moderate damage; (+) describes mild change; (−) indicates a lack of histologic change.

**Table 2:** Primers sequences of genes.

Target	Forward Primer	Reverse Primer
*β. Actin*	TCC TCC TGA GCG CAA GTA CTCT	GCT CAG TAA CAG TCC GCC TAGAA
*Bcl-2*	CAC CCC TGG CAT CTT CTC CTT	AGC GTC TTC AGA GAC AGC CAG
*NF-κB*	GTC TCA AAC CAA ACA GCC TCAC	CAG TGT CTT CCT CGA CAT GGAT
*Caspase-3*	AGT TGG ACC CAC CTT GTG AG	AGT CTG CAG CTC CTC CAC AT

## Data Availability

The data that support the findings of this study are available from the corresponding author upon reasonable request.

## Abbreviations

The following abbreviations are used in this manuscript:

5-FU	5-Fluorouracil
ADP	Adenosine diphosphate
AKI	Acute kidney injury
Bax	Bcl-2 Associated X-protein
BCL-2	B-cell lymphoma-2
BUN	Blood Urea Nitrogen
Alt	Alanine aminotransferase
AsT	Aspartate aminotransferase
ALP	Alkaline Phosphatase
Caspase-3	Cysteine aspartate-specific protease 3
CAT	Catalase
CONT	Control
DAB	3,3′-diaminobenzidine
DCT	Distal convoluted tubules
DLS	Dynamic light scattering
DNA	Deoxyribonucleic acid
ELISA	Enzyme-Linked Immunosorbent Assay
FT-IR	Fourier Transform Infrared
GAPDH	Glyceraldehyde-3-phosphate dehydrogenase
GPx	Glutathione Peroxidase
GSH	Glutathione synthetase
H&E	Hematoxylin and Eosin
HRP	Horseradish peroxidase
i.m	Intramuscular
IHC	Immunohistochemistry
IL-10	Interlukin-10
iNOS	Inducible Nitric Oxide Synthase
Keap-1	Kelch-like ECH-associated protein 1
KIM-1	Kidney Injury Molecule-1
LDH	Lactate Dehydrogenase
LPO	Lipid peroxidation
HO-1	Heme oxygenase-1
PAR	poly (ADP-ribose)
MDA	Malondialdehyde
NADH	Nicotinamide adenine dinucleotide
NF-κB	Nuclear Factor kappa B
NGAL	Neutrophil gelatinase-associated lipocalin
NO	Nitroxide
Nrf2	Nuclear factor erythroid 2-related factor 2
OS	Oxidative stress
PARP	Poly (ADP-ribose) polymerase
PCT	Proximal convoluted tubules
ROS	Reactive oxygen species
RT-qPCR	Quantitative reverse transcription polymerase chain reaction
EGCG	Epigallocatechin gallate
EGCG-SeNPs	Epigallocatechin gallate-Functionalized Selenium Nanoparticles
SEM	Standard Error of the Mean
SeNPs	Selenium Nanoparticles
SOD	Superoxide dismutase
TEM	Transmission Electron Microscopy
TNF-α	Tumor necrosis factor alpha
